# Variability and agreement of frailty measures and risk of falls, hospital admissions and mortality in TILDA

**DOI:** 10.1038/s41598-022-08959-7

**Published:** 2022-03-22

**Authors:** Dani J. Kim, M. Sofia Massa, Robert Clarke, Siobhan Scarlett, Aisling M. O’Halloran, Rose Anne Kenny, Derrick Bennett

**Affiliations:** 1 Clinical Trial Service Unit and Epidemiological Studies Unit (CTSU), Nuffield Department of Population Health, University of Oxford, Big Data Institute, Old Road Campus, Oxford, OX3 7LF UK; 2 The Irish Longitudinal Study on Ageing, Medical Gerontology, Trinity College, Dublin, Ireland; 3 The National Institute of Health (NIHR) Oxford Biomedical Research Centre (BRC), Oxford, UK

**Keywords:** Diseases, Health care, Medical research, Signs and symptoms

## Abstract

Little is known about the within-person variability of different frailty instruments, their agreement over time, and whether use of repeat assessments could improve the strength of associations with adverse health outcomes. Repeat measurements recorded in 2010–2011 (Wave 1) and 2012 (Wave 2) from The Irish Longitudinal Study on Ageing (TILDA) were used to classify individuals with frailty using the frailty phenotype (FP) and frailty index (FI). Within-person variability and agreement of frailty classifications were assessed using ANOVA and kappa (K) statistics, respectively. Associations of each frailty measure (wave 1, wave 2, or mean of both waves) with risk of falls, hospitalisations and all-cause mortality were assessed using logistic regression. Among 7455 individuals (mean age 64.7 [SD 9.9] years), within-person SD was 0.664 units (95% CI 0.654–0.671) for FP and 2 health deficits (SD 0.050 [0.048–0.051]) for FI. Agreement of frailty was modest for both measures, but higher for FI (K 0.600 [0.584–0.615]) than FP (K 0.370 [0.348–0.401]). The odds ratios (ORs) for all-cause mortality were higher for frailty assessed using the mean of two versus single measurements for FI (ORs for mortality 3.5 [2.6–4.9] vs. 2.7 [1.9–3.4], respectively) and FP (ORs for mortality 6.9 [4.6–10.3] vs. 4.0 [2.8–5.635], respectively). Frailty scores based on single measurements had substantial within-person variability, but the agreement in classification of frailty was higher for FI than FP. Frailty assessed using the mean of two or more measurements recorded at separate visits was more strongly associated with adverse health outcomes than those recorded at a single visit.

## Introduction

Frailty is a state of diminished resilience to external stressors and assessments of frailty are widely used in older people to predict adverse health outcomes. However, there is no consensus on the optimum instruments to assess frailty in older people and the available methods differ substantially in their conceptual background and content^1^. Importantly, there is no agreement on the precise definition of frailty and little is known about the within-person variability and agreement of frailty over time, and the extent to which use of different instruments to assess frailty in older people can reliably predict adverse health outcomes^2^.

The two most widely used instruments to classify frailty are the Frailty Phenotype (FP)^3^, which is a measure of incremental proportions of five physical characteristics, and the Frailty Index (FI)^4^, which is a measure of the cumulative proportions of up to 30 or more common diseases or causes of ill health (“deficits”). The FP and FI involve different exposures, but both involve a cumulative decline in multiple physiologic systems with increasing age. Irrespective of which instruments are used to quantify frailty, the syndrome is a continuum ranging from normal to pre-frail or frail, and strength of the associations with mortality and other adverse health outcomes vary with the severity of frailty^5,6^.

Previous studies comparing the predictive performance of different frailty measures have reported that while all frailty instruments were strongly associated with disease outcomes, FI had higher accuracy than FP for most disease outcomes^7,8^. However, the performance of all frailty instruments was limited irrespective of the instruments used to assess frailty^7–9^, prompting some to advocate the need for improvement in the instruments used to assess frailty.

While most observational studies of frailty in older people rely on measurements recorded at a single visit and have examined associations with disease outcomes after an interval of several years or decades, the extent to which a single measurement represents a stable measure of frailty for several years, and whether this can affect the prediction of disease outcomes, is unknown. Importantly, the severity of frailty fluctuates within individuals, partly due biological variability, onset or acute exacerbations of disease, changes in treatments, and effects of chance or random variability. Reliable estimates of associations of frailty with disease outcomes assessed using different instruments requires measurements of within-person variability in frailty, in individuals across multiple time points, for each frailty instrument. In the setting of substantial within-person variability (due to random error), the assessment of frailty based on a single measurement becomes imprecise, therefore the associations of frailty with disease outcomes may be substantially underestimated^10,11^.

The aims of the present report were: (i) to compare the within-person variability and agreement of frailty status when assessed using either FP or FI, and (ii) to compare the strength of the associations of falls, hospitalisation or death with frailty recorded using either FP or FI at single versus multiple visits in a population-based prospective study of adults aged 50 years and older.

## Methods

### Study population

The Irish Longitudinal Study on Ageing (TILDA) is a prospective study of 8175 community-dwelling participants aged ≥ 50 years at baseline and their younger spouses or partners (n = 329). Data were collected in sequential surveys (Wave 1 in October 2009–February 2011 and Wave 2 in February 2012–March 2013) by trained interviewers using a computer-assisted personal interview (CAPI). The questionnaires collected information on medical history, lifestyle factors, and social and economic factors. At Wave 1, participants were invited to undertake a nurse-led comprehensive health assessment at a dedicated centre (or in their own homes), in which participants had measurements of anthropometry, physical, cognitive, cardiovascular and visual function recorded, and a blood sample was collected for long-term storage^12,13^. All study methods were carried out in accordance with relevant guidelines and regulations. Ethics approval was granted by the Trinity College Research Ethics Committee and all participants provided written informed consent.

### Frailty measures

Frailty was classified using either the Phenotype of Frailty (FP)^3^ or Frailty Index (FI) (or accumulation of deficits)^4^ models. FP consists of five components: weakness, physical activity, walking speed, weight loss and exhaustion. These criteria, using methodology outlined by Fried et al.^3^, were used to identify participants with the lowest capacity for each component (Supplementary Table S1). FI was defined using 40 items according to the procedures outlined by Searle et al.^14^ that included self-reported, binary or ordinal health deficits covering multiple physiological systems including chronic diseases, limitations in mobility and basic and instrumental activities of daily living, cognitive function, depressive symptoms, sensory impairment and self-rated health (Supplementary Table S2). Frailty was studied as an ordinal score [0–5] for FP, as a ratio [0–1] for FI, and using categorical levels for both. Group levels (non-frail, pre-frail and frail) of the FP and FI were defined using well-established thresholds and applied to frailty measured at two sequential surveys (Wave 1 and Wave 2) and the mean of the values recorded at both surveys.

### Covariates

Participants were classified by sex as male or female. Highest education level had 8 categories: some primary (not complete), primary or equivalent, intermediate/junior/group certificate or equivalent, leaving certificate or equivalent, diploma/certificate, primary degree, postgraduate/higher degree or none. Participant’s social class categories had 7 categories: professional workers, managerial and technical, non-manual, skilled manual, semi-skilled, unskilled, or unknown/refused. Marital status had 6 categories: married, living with a partner as if married, single (never married), separated, divorced or widowed. Participants were defined as a smoker if they had ever smoked daily for a period of at least one year. Participants were defined as drinkers if their alcohol consumption exceeded the weekly limit of standard drinks defined by the government guidelines.

### Disease outcomes

Outcomes studied included: (i) overnight hospital admissions reported in both waves (or recurrent overnight hospital admissions); (ii) falls reported in both waves (recurrent falls); and (iii) all-cause mortality. Data on mortality were obtained from Irish General Register Office up to March 2018^15^. Falls and overnight hospital admissions occurring during the previous year were self-reported and obtained from the CAPI questionnaire. Recurrent outcomes, defined as participants reporting outcomes at both study waves, were used for comparisons of frailty measured at different time points.

### Statistical analyses

Measures of medical history, lifestyle and frailty measurements were recorded at each wave. The agreement between different measures of frailty at each wave was assessed using Cohen’s kappa^16^ for categorical exposures and Spearman’s correlation coefficients for continuous exposures. Kappa (K) scores ≥ 0.8^16^ or Spearman’s correlation of ≥ 0.6^17^ were interpreted as having moderate agreement.

#### Structural validity of frailty instruments

The structural validity (Supplementary Text S1) of each frailty model was assessed using factor analysis before the measurement properties of each construct were studied. We assessed the dimensionality (i.e. the internal relationships between the variables that represent the latent, unobservable, construct of interest) of each construct and the extent to which the individual components of frailty influenced the overall constructs. First, we performed exploratory factor analysis (EFA) to investigate the factor structure of the FP and FI using data from Wave 1 (EFA identifies this latent structure without imposing any preconceived structure), and then confirmatory factor analysis (CFA) was performed using Wave 2 to verify the hypothesised structure identified in the EFA^18^. EFA was performed using the weighted least squares method for factor extraction^19^, with oblique (promax) rotation^20^. The proportion of covariance explained by each factor^18^ and total variance explained by the factor structure were reported. CFA was performed in Wave 2 using weighted least square mean and variance adjusted (WLSMV) estimation^19^. Additional details of EFA and CFA are provided in Supplementary Text S2.

#### Imputation of missing data

The number of participants with missing values in frailty-related variables were recorded (Supplementary Table S3). Multiple imputation (MI) by Chained Equations was used to impute missing data^21^. We carried out 20 imputations^22^ with 10 iterations using the classification and regression trees method^23^. The following variables were imputed: age, sex, smoker, area of residence, social class, education level, living arrangement, marital status, medical history, disability information, hospital use, cognition, number of falls, attendance of health assessment centre at baseline and FP variables. Margin plots to assess the missing-at-random assumption are shown in Supplementary Figs. S1–S3.

#### Within- and between-person variability of frailty measurements

We estimated the within-person variability of frailty measures using one-way analysis of variance (ANOVA) and standard errors of measurement (SEM). The analysis included estimates of the minimal detectable change (MDC) and minimally importance change (MIC) in frailty measures. ANOVA was used to estimate both within- and between-person standard deviation (SD)^24^, with higher SDs indicating less consistency in frailty scores. SEM^25,26^, which estimates the variability of the errors of measurement in an individual around their “true” score, were estimated using the following equation: SD of the test × √(1 − correlation coefficient). The correlation coefficient for the FP and FI was determined using Kendall’s W and Lin’s concordance correlation coefficients, respectively. Since the units of SEM and the original test scores were identical, SEM were used to assess precision of single measurements of test scores. A higher SEM estimate indicates a higher level of within-person variability and poor precision. SEM was also used to estimate the MDC^27^, which is the minimal change required to distinguish a true change in performance from a change due to errors in measurement, using the following equation: 1.96 × √2 × SEM. The MDC_95_ provides an estimate of error around repeated measures. Overall, 95% of the observed differences between pairs of observations will be smaller than the MDC values given that there are no true differences indicating any changes greater than MDC_95_ can be confidently attributed to real change. MIC^28^ reflects the smallest change in test scores detectable as clinically meaningful improvement or deterioration in health by individuals or clinicians and was estimated by standard methods. Using self-reported health status as the anchor^27,29,30^, we defined MIC as two or more units decline in self-reported health status (5-levels in total: Excellent, Very good, Good, Fair, and Poor). We used the change difference method^31^ which defined MIC as the difference between the average frailty score change of those reporting a transition in the anchor status (a deterioration in self-reported health) and those reporting no change^30,31^. By comparing the magnitude of the MIC and MDC, we determined whether any clinically meaningful changes can be distinguished from within-person variability (i.e. MIC is larger than MDC)^32^. Bootstrapping was used to calculate 95% confidence intervals using 1000 samples^33^.

#### Within-person agreement in frailty measurements

Agreement in the classification of frailty status within individuals was assessed using intra-class kappa scores^34,35^. Bland–Altman (BA) plots, plots of the intra-individual means of the sequential measurements and their corresponding standard deviations were plotted to demonstrate the strength and pattern of agreement for continuous scores^24,36,37^. Additional analyses assessed the reliability in age-specific groups.

#### Associations with recurrent falls, hospital admission and all-cause mortality

Logistic regression was used to estimate the associations of adverse health outcomes and all-cause mortality with each frailty measure after adjustment for age, sex, education level, social class, marital status, smoking status, and alcohol consumption. Odds ratios (ORs) for categorical levels of frailty were used to compare associations with the mean of the two repeated measures of frailty versus a single measurement. The area under the receiver-operating characteristic curves (AUC) were estimated for each measure of continuous frailty scores^38^. An AUC ≥ 0.70 indicates good discriminatory power^32^. All analyses were performed using R, version 4.0.2 (R Core Team, 2021).

## Results

Among a total of 8504 participants included at Wave 1, 7455 were followed-up, 1011 were lost to follow-up and 208 had died at Wave 2. At baseline, participants had a mean (SD) age of 63.1 (10.2) years; 55.6% were female; 70% were married; and over one-quarter were manual workers (Table 1). Overall, demographic factors, lifestyle or medical history variables did not change appreciably within 2 years, but participants at Wave 2 were more likely than those at Wave 1 to use 5 or more medications daily (28% vs 20%), have fewer smokers (16% vs 18%) and more likely to have attended the baseline health centre assessment (68% vs 62%). The median FP (0) and FI (0.12/0.13) remained constant over 2 years. Participants who were lost to follow-up had a more adverse health status at baseline than those who were followed-up at Wave 2 (Supplementary Table S4).

**Table 1:** Selected characteristics of study participants at Waves 1 and 2.

	Wave 1	Wave 2	Lost to follow-up or death
Mean (SD), median (IQR) or n (%)	(n = 8504)	(n = 7455)	(n = 1219)
**Demographic**			
Age, years	63.1 (10.2)	64.7 (9.9)	65.4 (11.4)
Sex (female)	4724 (55.6%)	4149 (55.7%)	670 (55%)
Education			
Primary	2975 (35%)	2469 (33.9%)	506 (41.5%)
Secondary	3431 (40.3%)	2983 (40.9%)	448 (36.8%)
Tertiary	1818 (21.4%)	1617 (22.2%)	201 (16.5%)
Married	5966 (70.2%)	5220 (70%)	796 (65.3%)
Lives alone	1822 (21.4%)	1510 (20.7%)	312 (25.6%)
Occupation			
Manual worker	2341 (27.5%)	2665 (35.7%)	410 (33.6%)
Non-manual worker	6163 (72.5%)	4790 (64.3%)	809 (66.4%)
**Lifestyle/medical history**			
BMI, kg/m^2^	28.7 (5.1)	27.1 (4.8)	28.6 (5.1)
Smoker	1564 (18.4%)	1168 (15.7%)	300 (24.6%)
Drinker^a^	453 (5.3%)	402 (5.5%)	51 (4.2%)
Depressed	809 (9.5%)	638 (8.6%)	135 (11.1%)
MMSE errors	1.7 (2.2)	1.5 (2.2)	2.7 (3)
Baseline health assessment			
Health centre	5275 (62%)	4927 (67.6%)	348 (28.5%)
Home	875 (10.3%)	721 (9.9%)	154 (12.6%)
Missing	2354 (27.7%)	1637 (22.5%)	717 (58.8%)
Prior CVD^b^	1704 (20%)	1584 (21.2%)	255 (20.9%)
Prior cancer	522 (6.1%)	566 (7.6%)	97 (8%)
Diabetes	641 (7.5%)	610 (8.2%)	116 (9.5%)
Polypharmacy (≥ 5 medications)	1703 (20%)	2065 (27.7%)	260 (21.3%)
Falls in the past year (ever)	1640 (19.3%)	1652 (22.2%)	231 (18.9%)
Overnight hospitalisation in the past year (ever)	1085 (12.8%)	1047 (14%)	180 (14.8%)
**Frailty measurements**			
Grip strength, kg	26 (9.9)	29.4 (10.8)	25.3 (10)
Timed-Up-and-Go, seconds	9.1 (3.7)	9.5 (3.8)	10.3 (4.6)
IPAQ-SF, kcal/week	2274 (796.1–5488.1)	2079 (716.1–5196.8)	1716.9 (492.2–4299)
Frailty phenotype (FP, 0–5)			
Count	0 (0–1)	0 (0–1)	1 (0–2)
Non-frail	3087 (36.3%)	3308 (44.4%)	204 (16.7%)
Pre-frail	2492 (29.3%)	2829 (37.9%)	206 (16.9%)
Frail	374 (4.4%)	476 (6.4%)	64 (5.3%)
Missing	2551 (30%)	842 (11.3%)	745 (61.1%)
Frailty Index (FI, 0–1)			
Score	0.12 (0.06–0.2)	0.13 (0.07–0.22)	0.13 (0.07–0.22)
Non-frail	3576 (42.1%)	2803 (37.6%)	479 (39.3%)
Pre-frail	3517 (41.4%)	3265 (43.8%)	490 (40.2%)
Frail	1411 (16.6%)	1387 (18.6%)	250 (20.5%)

Continuous data presented as mean (SD) for age, BMI, grip strength and TUG, or median (IQR) for MMSE errors, frailty scores/count and IPAQkcal.

Baseline data reported for lives alone, education level, and prior CVD for all groups due to data availability.

^a^Drinkers consuming levels above the weekly limit on standard drinks (1/2 pint of beer or a glass of wine) according to the government guidelines (> 21 for men; > 14 for women).

^b^CVD defined as any of the following present: angina, heart attack, diabetes, stroke, transient ischaemic attack, or heart murmur.

*BMI* body mass index, *CVD* cardiovascular diseases, *FI* frailty index, *FP* frailty phenotype, *MMSE* Mini-Mental State Examination, *IPAQ-SF* International Physical Activity Questionnaire-Short Form.

The FP and FI models had good structural validity at separate time-points, which provides further support for the results involving repeat measurements of frailty in the present paper. In brief, the EFA in Wave 1 identified a 2- and 4-factor solution for the FP and FI, respectively (Supplementary Table S5). The FI’s factor solution accounted for 53% of the total variance in the FI variables, while FP’s solution accounted for 31% of the total variation in the FP components. For both models, the factors related to physical functioning (walk time for FP and instrumental activities of daily living, IADLs, for FI) were most important factors explaining the latent frailty construct. A consistent factor solution was found for the FP (Supplementary Fig. S4) and FI (Supplementary Fig. S5) through CFA in Wave 2.

Agreement between the frailty instruments was only modest at both waves (weighted kappa: 0.394 [95% CI 0.386–0.400] at Wave 1 and 0.453 [0.447–0.459] at Wave 2; Spearman’s rho: 0.448 [0.428–0.467] at Wave 1 and 0.520 [0.502–0.539] at Wave 2) (Table 2). Fewer participants were defined as being frail by FP (Table 1), which also tended to classify participants as being less frail than by FI (Table 2).

**Table 2:** Agreement between classification of individuals with frailty using the frailty phenotype and frailty index at Waves 1 and 2.

		Frailty phenotype (wave 1)			Agreement between FP & FI	
					Spearman’s rho (95% CI)	Weighted kappa (95% CI)
		Non-frail	Pre-frail	Frail		
Frailty	Non-frail	1725	769	22	0.448 (0.428–0.467)	0.394 (0.386–0.400)
Index	Pre-frail	1204	1204	111		
(Wave 1)	Frail	158	519	241		

		Frailty phenotype (wave 2)			Spearman’s rho (95% CI)	Weighted kappa (95% CI)
		Non-frail	Pre-frail	Frail		
Frailty	Non-frail	1836	793	22	0.520 (0.502–0.539)	0.453 (0.447–0.459)
Index	Pre-frail	1329	1446	173		
(Wave 2)	Frail	143	590	281		

Data on variability in frailty measurements are shown in Table 3. At baseline, the mean (SD) scores of FP and FI were 0.756 (0.969) and 0.145 (0.108), respectively; but both had comparable magnitude of variation for Wave 2 and for the mean of both waves. The between-person standard deviation (SD) obtained from ANOVA was 1.247 (1.243–1.257) and 0.148 (0.145–0.151) for FP and FI, respectively. Hence, over half of participants’ scores were within the ranges of 0–1 and 0.05–0.19 for the FP and FI, respectively. Conversely, the within-person SD in frailty were 0.664 (0.654–0.671) and 0.050 (0.048–0.051) for FP and FI, respectively (SEM was 0.473 [0.470–0.478] and 0.048 [0.047–0.050], respectively), which indicates that individual’s observed scores on a single administration were within ± 0.5/0.7 units (FP) or 2 (2/40 = 0.05) deficits (FI) of their true values. The MDC_95_ and MIC values (see Supplementary Table S6 for calculation of MIC) were 1.310 (1.301–1.324) and 0.099 (0.035–0.154) for FP and 0.134 (0.131–0.138) and 0.044 (0.036–0.054) for FI, respectively. These results suggest that any differences less than 1.31 (FP) and 0.13 (or 5 deficits, FI) between two assessments could be expected by chance alone, so while an increase in scores by 0.10 (FP) and 2 deficits (FI) from Wave 1 and Wave 2 might represent a detectable deterioration in self-rated health, it cannot be distinguished from chance alone.

**Table 3:** Within-person variability in the frailty phenotype and frailty index.

	Frailty phenotype	Frailty index
Mean (SD) wave 1	0.756 (0.969)	0.145 (0.108)
Mean (SD) wave 2	0.825 (1.433)	0.159 (0.112)
Mean (SD) wave 1 and 2	0.791 (1.212)	0.152 (0.111)
Within-person SD^a^	0.664 (0.654–0.671)	0.050 (0.048–0.051)
Between-person SD^a^	1.247 (1.243–1.257)	0.148 (0.145–0.151)
Correlation coefficient^b^	0.752 (0.748–0.755)	0.800 (0.791–0.808)
Standard error of measurement	0.473 (0.470–0.478)	0.048 (0.047–0.050)
Minimally detectable change	1.310 (1.301–1.324)	0.134 (0.131–0.138)
Minimally important change*	0.099 (0.035–0.154)	0.044 (0.036–0.054)

^a^Within- and between-person variance was calculated using the one way analysis of variance of method^24^.

^b^Correlation coefficients are Kendall’s W and Lin’s concordance correlation coefficient for FP and FI, respectively.

*See Supplementary Table S6 for more detail on how MIC was calculated.

**Figure 1:**
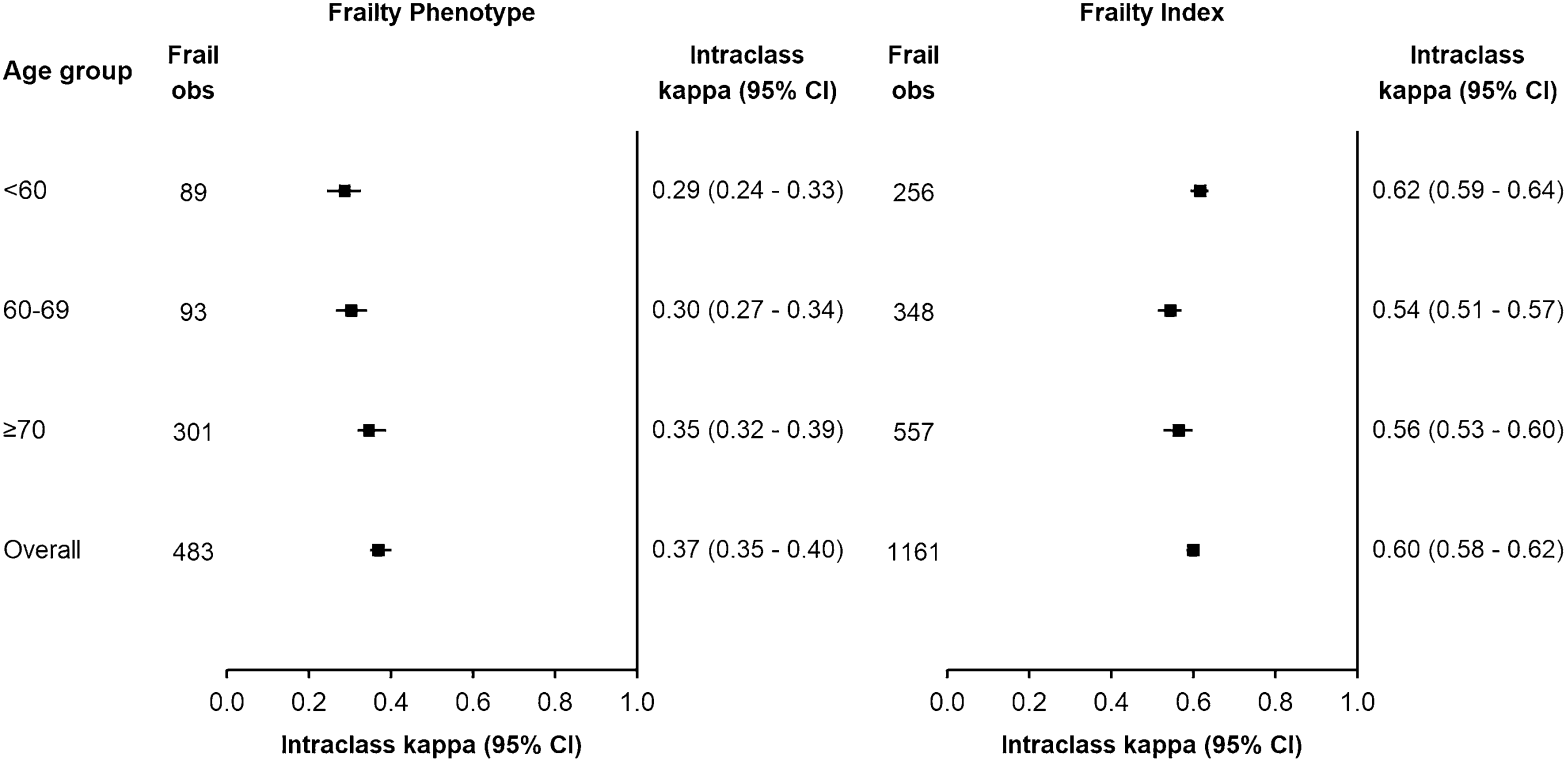
Agreement in classification of frailty over 2 years using frailty phenotype and frailty index, overall and by age group. Frail refers to the number of frail participants at Wave 1. Total participants at Wave 1 by age group were 3154 (< 60), 2265 (60–69); and 1866 (≥ 70).

Figure 1 demonstrates a higher level of agreement in frailty status over 2 years by FI over FP when estimated using the kappa statistics (K statistic: 0.600 [0.584–0.615] vs. 0.370 [0.348–0.401], respectively). The distribution of changes in scores (Supplementary Fig. S6) demonstrated minimal changes in frailty status for most participants. The BA plot shows some heterogeneity in agreement by severity of FI scores, suggesting that participants with higher average FI scores tended to improve over time (Supplementary Fig. S7).

**Figure 2:**
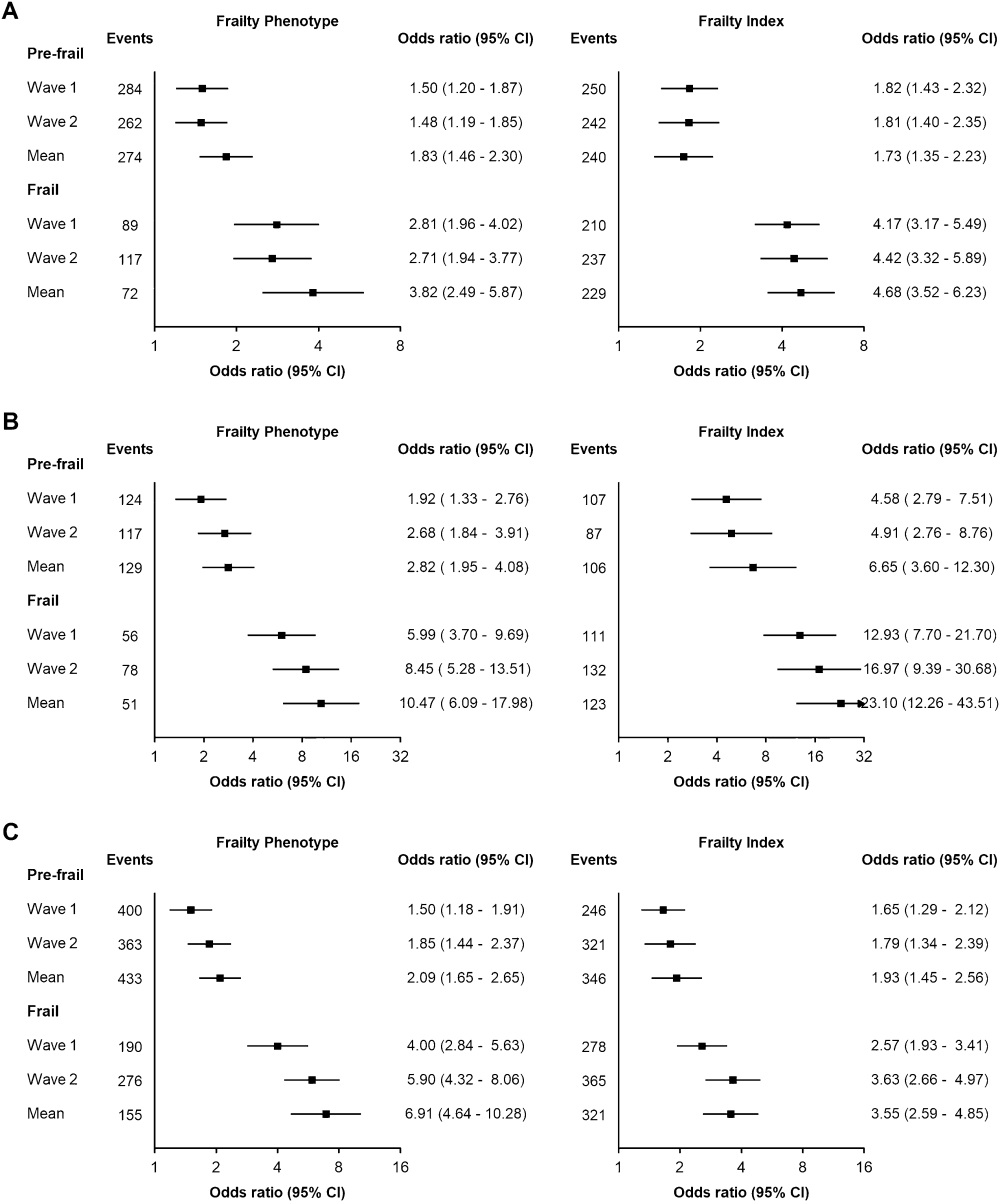
Associations of frailty phenotype and frailty index with recurrent falls (**A**) and overnight hospital stay (**B**) and all-cause mortality (**C**). Odds ratios (OR) were adjusted for age, sex, education level, social class, marital status, smoking status, and alcohol drinking frequency at baseline. Reference level was non-frail group for each frailty definition. Recurrent outcomes were defined as experiencing a disease outcome at Waves 1 and 2.

Both frailty measures were strongly associated with higher risks of adverse health outcomes (Fig. 2). The ORs associated with higher levels of frailty classified by the FI were greater than those classified by FP for recurrent falls and hospital admission, but not for all-cause mortality. For both measures, frailty classification based on the mean of repeat frailty measurements were more strongly associated with adverse health outcomes than scores on measurements based on single measurements. For FP and FI, the strength of associations of frailty with recurrent hospitalisation were nearly twofold greater using the mean of measures recorded at two visits vs a single visit. For FI, Wave 2 frailty measurements were most strongly associated with all-cause mortality. The AUCs were also higher for FI than FP and comparable irrespective of the number of frailty measurements used (single vs. mean) (Supplementary Table S7).

## Discussion

Frailty scores for individuals based on measurements recorded at a single visit were associated with high within-person variability. Although frailty scores were stable over 2 years for most participants, estimates were associated with a within-person variability of about 0.7 units for FP and 2 deficits for FI. Overall, the within-person agreement of frailty status when assessed by either FP or FI was only modest and clinically meaningful changes in self-reported health status over 2 years were approximately one-tenth unit for FP and 2 deficits for FI in adults aged 50 years and older. Importantly, the strengths of the associations of adverse health outcomes with frailty measurements based on two visits with a 2-year interval were almost twofold greater than those based on the single, baseline visit.

Within-person variability may arise from measurement error due to an imperfect instrument or operator and intra-individual variation in components used to classify frailty may vary over time, reflecting the dynamic processes of disease and disability^39^, or the influence of clinical and lifestyle interventions^40^. The findings of the present study are consistent with those of a previous study^41^ that also reported estimates for intra-individual variability of 0.04/0.05 for FI using mixed-effects regression models, which allowed the modelling of the mean (‘location’) and variation (‘scale’) of frailty simultaneously on data with up to five repeated measurements. The findings are also consistent with those estimates for MIC (anchor-based) in a Korean study of older people, which were 0.030 for FI and 0.097 for FP^42^. Consistent with Thompson et al.^43^, we also demonstrated that more recent measurements of frailty were more strongly associated with adverse health outcomes with comparable discrimination compared with those estimated using baseline measures. Importantly, we demonstrated that combining the baseline and repeat measurements yielded stronger associations with adverse health outcomes compared with those based on single measurements. The apparent increase in strength of associations may reflect some correction for the variability associated with single measurements in frailty and the persistence of frailty over time. For FI, it was the most recent value that was most strongly associated with all-cause mortality, which may reflect the cumulative nature of deficits in frailty that incorporate values from previous measures.

The lack of consensus on the optimum instruments to assess frailty is a challenge for clinicians and researchers to select frailty instruments, and hence, different frailty instruments are typically used interchangeably despite the substantial heterogeneity in their content and limited agreement in such measures within individuals. To evaluate optimum measures for assessment of frailty, we compared models based on their variability and ability to predict adverse health outcomes^44^. By providing estimates of within-person variability in the same individuals for both FP and FI, we demonstrated that the FI had higher levels of agreement at 2 years between measures and was a stronger predictor of adverse health outcomes. The structural validity analyses also provided additional evidence in favour of F1 over FP. The most important deficits accounting for the latent frailty structure were those related to instrumental activity of daily living (IADLs), which may explain the enhanced stability. However, for both the FP or FI instruments, the absolute level of variability was high, and hence, limiting the ability to detect clinically meaningful changes.

Frailty instruments are routinely used in clinical practice^45^ in order to identify individuals at high-risk of a wide range of adverse health outcomes. In the present report, we demonstrated high variability in both measures of frailty. The available evidence demonstrated suboptimal predictive accuracy of these widely used frailty scores to predict adverse health outcomes^46^. Despite accumulating evidence of such limitations, no major modifications to the FP and FI have been routinely adopted to date. The findings of the present study demonstrate that combining replicate measurements may strengthen the associations of frailty with adverse health outcomes, and may yield discernible health benefits by improving the accuracy to identify older people at higher risks of adverse health outcomes. For the FP, the combined mean score had a twofold greater strength of association than baseline values alone with higher AUCs. Hence, in settings where electronic records are widely used it should be relatively simple to implement an assessment of frailty using measurements recorded at multiple time points. Attention to detail about within-person variability in frailty measures and comparative performance of different instruments to quantify frailty is important when assessing the determinants of frailty or prognosis following a diagnosis of frailty in population studies and in clinical practice. The present study also provides support for an emerging consensus that different frailty instruments measure different constructs^47^. FP measures a progressive state of low energy, sarcopenia and low strength^48^, but FI measures the loss of physiological reserve through lower redundancy^49^, and hence it may be prudent not to use these different measures interchangeably.

The present report had several strengths, including longitudinal data from a large cohort study, a comprehensive comparison of several understudied measurement properties (variability and structural validity) of frailty measures^2,50–52^, and multiple imputation to minimise the impact of missing data to compute FP on the comparisons between frailty measures. However, this study also had several limitations. First, some differences in the way FP variables were measured across TILDA waves meant more accurate measurements at baseline versus the second wave, such as grip strength (Supplementary Table S1), which may have resulted in an over-estimation of the variability of FP^53^. Second, the large number of participants lost to follow-up, who differed from those that completed the study, may have underestimated the associations of both frailty measures with adverse health outcomes. Third, interventions for frailty issues may have occurred during the interim, but this information was unavailable and could not be included in the present analyses. Finally, it is possible that the estimates of within-person variability based on paired measurements of frailty over a 2-year interval may have been underestimated and more frequent measurements over a longer period may be required to assess their impact on prediction of adverse health outcomes over a longer follow-up period. However, it is likely that there may be even more extreme within-person variability associated with longer intervals between measurements.

## Conclusions

The present report demonstrated that the FI was a more stable measure of frailty than FP and a more accurate predictor of adverse health outcomes in adults aged 50 years and older, but irrespective of which instrument is used to assess frailty, a single measurement of frailty was associated with substantial within-person variability. However, use of replicate measurements of frailty recorded at separate visits compared to single assessments, particularly at baseline, may strengthen the associations of frailty with adverse health outcomes in observational studies.

## Supplementary Information

Supplementary Information.
