# Supplementary material for: Variability and agreement of frailty measures and risk of falls, hospital admissions and mortality in TILDA
Source: Sci Rep. 2022 Mar 22;12:4878. doi: 10.1038/s41598-022-08959-7 (PMC8940970; doi:10.1038/s41598-022-08959-7)
Supplement: Supplementary file 1 — Supplementary Information. [file 41598_2022_8959_MOESM1_ESM.docx]

Supplementary Material

**Variability and agreement of frailty measures and risk of falls, hospital admissions and mortality in TILDA**

Dani J. Kim MSc^a^, M. Sofia Massa PhD^a^, Robert Clarke FRCP^a*^, Siobhan Scarlett PhD^b^, Aisling M. O’Halloran PhD^b^, Rose Ann Kenny FRCPI^b^, Derrick Bennett PhD^ac^

^a^Clinical Trial Service Unit and Epidemiological Studies Unit (CTSU), Nuffield Department of Population Health, University of Oxford, Oxford, UK.

^b^The Irish Longitudinal Study on Ageing, Medical Gerontology, Trinity College, Dublin, Ireland.

cThe National Institute of Health (NIHR) Oxford Biomedical Research Centre (BRC).

*Corresponding author’s email: Robert.clarke[@ndph.ox.ac.uk](mailto:jennifer.carter@ndph.ox.ac.uk)

Table of Contents

[Table S1. Operationalisation of the Phenotype of Frailty model 3](#_Toc88571702)

[Table S2. Components of the 40-item frailty index 4](#_Toc88571703)

[Text S1. Glossary of key terms 6](#_Toc88571704)

[Text S2. Structural validity analysis 7](#_Toc88571705)

[Table S3. Number of missing values in frailty-related variables 8](#_Toc88571706)

[Figure S1. Margin plots between grip strength and other participant characteristics at Wave 1 to assess MAR assumption 9](#_Toc88571707)

[Figure S2. Margin plots between walk time and other participant characteristics at Wave 1 to assess MAR assumption 10](#_Toc88571708)

[Figure S3. Margin plots between physical activity and other participant characteristics at Wave 1 to assess MAR assumption 11](#_Toc88571709)

[Table S4. Baseline characteristics of participants followed-up versus participants lost to follow-up 12](#_Toc88571710)

[Table S5. Exploratory Factor Analysis (EFA) results of the frailty phenotype and frailty index 13](#_Toc88571711)

[Figure S4. Factor plot of a correlated 2-factor Confirmatory Factor Analysis (CFA) model of Frailty Phenotype (FP) 14](#_Toc88571712)

[Figure S5. Factor plot of a correlated 4-factor Confirmatory Factor Analysis (CFA) model of Frailty Index (FI) 15](#_Toc88571713)

[Figure S6. Distribution of the change in frailty phenotype count (A) and frailty index score (B) between Waves 1 and 2 16](#_Toc88571714)

[Figure S7. Bland-Altman plot for the frailty index score 17](#_Toc88571715)

[Table S6. Average changes in frailty phenotype count and frailty index score between Waves 1 and 2 by level of change in self-reported health status over the same period 18](#_Toc88571716)

[Table S7. Discrimination of the frailty phenotype and frailty index for recurrent falls and overnight hospital stay and all-cause mortality 19](#_Toc88571717)

[References 20](#_Toc88571718)

# Table S1. Operationalisation of the Phenotype of Frailty model

| **FP component** | **Definition using TILDA variables** | **Criteria** |
| --- | --- | --- |
| Weakness | Sex- and BMI-adjusted grip-strength measured on the dominant hand using dynamometer. Weight and height were measured using standardised procedures during home and health centre assessments. | Lowest quintile |
| Physical activity | Sex-adjusted kilocalories (kcals) from the International Physical Activity Questionnaire – Short Form [IPAQ-SF]). | Lowest quintile |
| Slow walking speed | Sex- and height-adjusted time taken in seconds to perform the Timed-Up-and-Go (TUG) task. | Slowest quintile |
| Unintended weight loss | Ascertained by the question “In the past year have you lost 10 pounds (4.5kg) or more in weight when you were not trying to.” | Answer “Yes” |
| Exhaustion | Ascertained using two items from the 20-item Centre for Epidemiological Studies Depression (CES-D) scale. Participants were asked how often they felt that “I could not get going” and “I felt that everything I did was an effort”. | A response of “moderate amount/all of the time” to either question |

Table adapted from [1,2]

Participants with 0, 1-2, and ≥3 of these criteria were defined as non-frail, pre-frail, and frail, respectively. The cut-off points of the weakness, physical and slow walking speed criteria were based on the TILDA population following the methodology of Fried et al. (2001). Importantly, there were only minor differences in the way some of the variables were measured between Wave 1 (home and/or health assessment centre) and Wave 2 (home assessment only) of TILDA. Wave 1 contains objective height and weight data compared to self-reported data in Wave 2. In Wave 1, two measurements of grip strength were taken, which were then used to compute mean grip strength, compared to a single measure in Wave 2.

# Table S2. Components of the 40-item frailty index

| **Variables in TILDA** | **Cut-points** |
| --- | --- |
| 1. Difficulty walking 100 m | Yes=1; No=0 |
| 2. Difficulty jogging 1.5 km | Yes=1; No=0 |
| 3. Difficulty rising from chair | Yes=1; No=0 |
| 4. Difficulty climbing several flights of stairs | Yes=1; No=0 |
| 5. Difficulty climbing one flight of stairs | Yes=1; No=0 |
| 6. Difficulty stooping, kneeling, or crouching | Yes=1; No=0 |
| 7. Difficulty reaching above shoulder height | Yes=1; No=0 |
| 8. Difficulty pushing/pulling large objects | Yes=1; No=0 |
| 9. Difficulty lifting/carrying weights ≥10 lb | Yes=1; No=0 |
| 10. Difficulty picking up coin from table | Yes=1; No=0 |
| 11. Difficulty preparing a hot meal | Yes=1; No=0 |
| 12. Difficulty with household chores | Yes=1; No=0 |
| 13. Difficulty shopping for groceries | Yes=1; No=0 |
| 14. Feeling lonely | Rarely or none of the time=0;  Some or a little of the time=0.33;  Occasionally or a moderate amount of time=0.66;  All of the time (5-7 days)=1 |
| 15. Poor self-rated physical health | Excellent=0; Very good=0.25; Good=0.5;  Fair=0.75; Poor=1 |
| 16. Poor self-rated vision | Excellent=0; Very good=0.25; Good=0.5;  Fair=0.75; Poor=1 |
| 17. Poor self-rated hearing | Excellent=0; Very good=0.25; Good=0.5;  Fair=0.75; Poor=1 |
| 18. Difficulty following a conversation with one person | None=0; Some=0.5; Much/Impossible=1 |
| 19. Daytime sleepiness | Would never doze=0;  Slight chance of dozing=0.33;  Moderate chance of dozing=0.66;  High chance of dozing=1 |
| 20. Polypharmacy (≥5 medications) | Yes=1; No=0 |
| 21. Intrusive pain | Yes=1; No=0 |
| 22. Knee pain | Yes=1; No=0 |
| 23. Urinary incontinence | Yes=1; No=0 |
| 24. Hypertension or high blood pressure | Yes=1; No=0 |
| 25. Angina | Yes=1; No=0 |
| 26. Heart attack | Yes=1; No=0 |
| 27. Diabetes | Yes=1; No=0 |
| 28. Stroke | Yes=1; No=0 |
| 29. Transient ischemic attack | Yes=1; No=0 |
| 30. High cholesterol | Yes=1; No=0 |
| 31. Irregular heart rhythm | Yes=1; No=0 |
| 32. Other cardiovascular disease | Yes=1; No=0 |
| 33. Cataracts | Yes=1; No=0 |
| 34. Glaucoma | Yes=1; No=0 |
| 35. Age related macular degeneration | Yes=1; No=0 |
| 36. Chronic lung disease | Yes=1; No=0 |
| 37. Arthritis | Yes=1; No=0 |
| 38. Osteoporosis | Yes=1; No=0 |
| 39. Cancer | Yes=1; No=0 |
| 40. Varicose ulcer | Yes=1; No=0 |

Table from supplementary material in [3]

The 40 deficits were computed using self-reported variables from the TILDA computer-assisted personal interview (CAPI) questionnaire which covered a range of domains including ability in activities of daily living (ADL), chronic diseases, medications, and cognition. Binary deficits were coded as present (1) or absent (0) and ordered categorical deficits were coded as a fraction proportional to the number of responses. The variables were summed and divided by 40, which produced the FI score between 0 and 1. Scores of <0.10, ≥0.10 to <0.25 and ≥0.25 were classified as non-frail, pre-frail and frail, respectively.

# Text S1. Glossary of key terms

**Validity** assesses whether an instrument measures the construct it purports to measure.[4]

**Structural validity** is the degree to which measurements of an instrument are an accurate reflection of the dimensionality of the construct to be measured.[5] An instrument’s dimensionality concerns the internal relationships between variables. This can be evaluated by **factor analysis**, a statistical technique for data reduction aimed at attributing sets of observed variables to one or more latent variables.[6]

**Standard error of measurement** is an estimate of 1 SD of the error associated with a single measurement, i.e. a 68% likelihood that the person’s true score is within 1±SEM of the observed score.[9]

**Minimally Detectable Change** is the minimum amount of change in a measurement tool that is required to distinguish a true performance change from a change due to chance variability.[10]

**Minimally Important Change** is the smallest measured change in the score of a measurement tool that is perceived as being important by patients and/or clinicians.[11]

# Text S2. Structural validity analysis

Structural validity was assessed using factor analysis, a statistical method that describes the shared variability among the observed, correlated variables that may represent a smaller number of unobserved, latent variables or factors.[12]

Further details about the Exploratory Factor Analysis, a form of factor analysis used to identify the underlying relationships between of a measured set of variables:[12]

1. The factorability of the FP and FI instruments was assessed using the Kaiser-Meyer-Olkin measure of sampling adequacy (KMO test)[13] and Bartlett’s test of sphericity [14] where KMO>0.60 and a significant Bartlett’s test indicate suitability for FA.
2. The number of factors extracted was based on the parallel test [15] and interpretability of the factor solution.[16–18]
3. Factor loadings, representing the strength of the association between the variable and the factor,[12] were also examined. We removed variables with a factor loading less than 0.3 [16,19] and cross-loading items (when the difference between the item that load onto multiple factors and the highest loading item is less than 0.2).[18,20]

Further details about the Confirmatory Factor Analysis, a form of factor analysis used to test whether the data fit a hypothesised measurement model:[12]

1. Model fit was assessed using the Comparative Fit Index (CFI),[21] Tucker-Lewis Index (TLI),[22] and the Root Mean Square Error of Approximation (RMSEA).[23] The criteria for adequate fit were: CFI≥0.95, TLI≥0.95 and RMSEA≤0.06.[24,25]

# Table S3. Number of missing values in frailty-related variables

| **Variables** | **% missing in Wave 1**  **(N=8504)** | **% missing in Wave 2 (N=7455)** |
| --- | --- | --- |
| Height | 27.9 | 2.2 |
| Weight | 27.9 | 3.5 |
| Grip strength | 28.7 | 6.5 |
| Physical activity | 28.5 | 4.3 |
| Energy | - | 1.1 |
| Time-up-and-go | 28.5 | 3.8 |

We explored whether these missing values could be explained by any other observed variables through a visual inspection of missing data (Figures S1-3).

Figures S1-S3 (next page) are margin plots, which are scatter plots between two continuous variables that display non-missing values in blue and missing values in red. In addition, the distributions of the values are displayed as box plots (on the extreme left) by whether the other variable is observed (left blue box) or unobserved (left red box). If distributions of values are different by whether data is missing or not, then the Missing-at-Random (MAR) assumption is plausible. Missingness patterns for Wave 2 are not shown, but were similar to Wave 1.


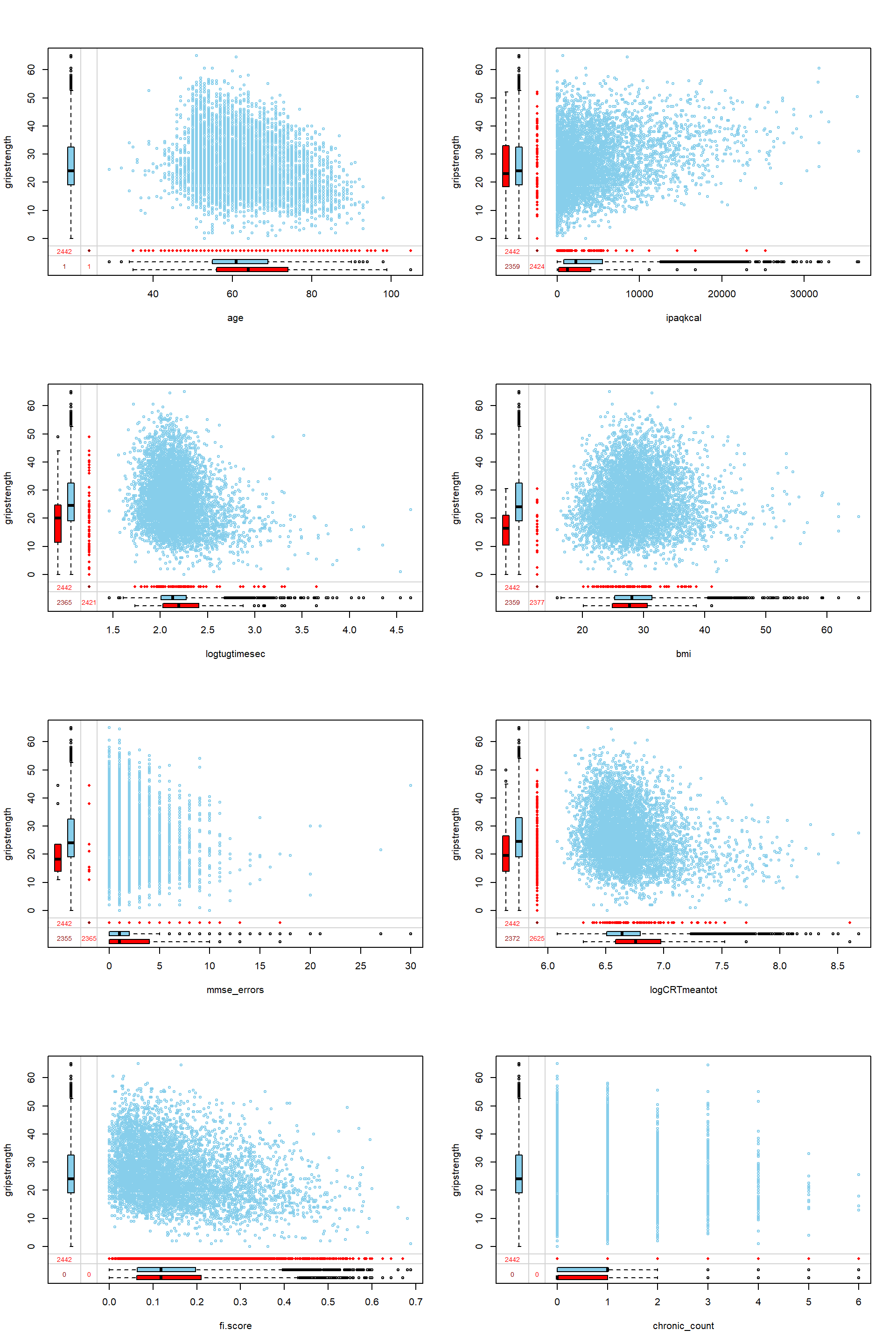
Figure S1. Margin plots between grip strength and other participant characteristics at Wave 1 to assess MAR assumption

At Wave 1, 2442 participants were missing grip strength. The distribution of grip strength was significantly lower for participants with values missing on Timed-Up-and-Go (TUG) time, BMI, the number of MMSE errors, and mean CRT (left red boxes) compared to participants where these variables were present (left blue boxes), suggesting that the missingness of grip strength might be dependent on these variables. Those with missing grip strength were also older (‘age’ p<0.001), slower i.e. higher TUG time (‘logtugtimesec’ p=0.02), inactive (‘ipaqkcal’ p<0.001), and have poor cognitive score (‘mmse_errors’ p=0.03). P-values refer to t-test or Wilcox test.

# Figure S2. Margin plots between walk time and other participant characteristics at Wave 1 to assess MAR assumption


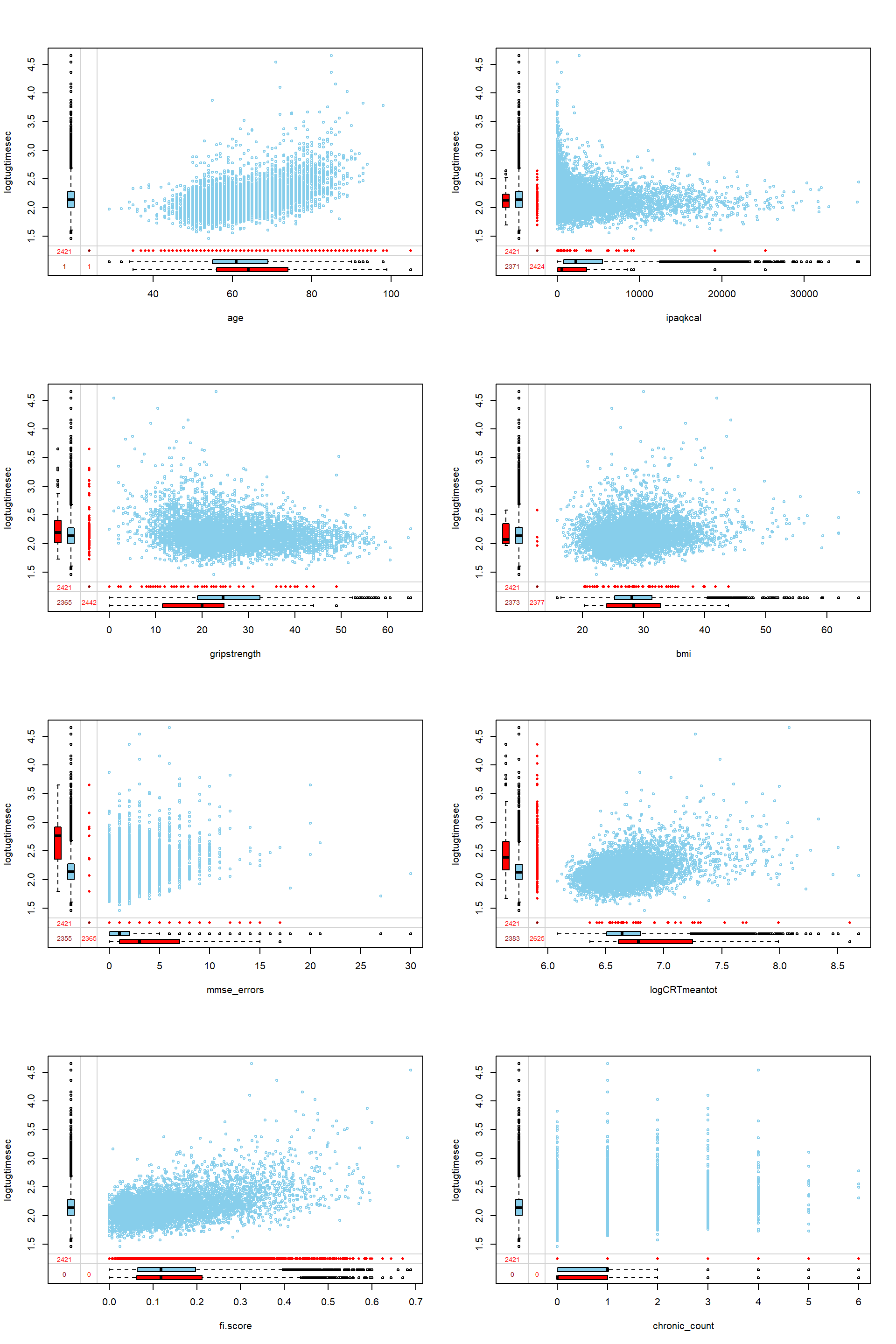


At Wave 1, 2421 participants were missing Timed-Up-and-Go (TUG) time. The distribution of TUG time was significantly higher for participants with values missing on the number of MMSE errors and mean CRT (left red boxes) compared to participants where these variables were present (left blue boxes), suggesting that the missingness of TUG time might be dependent on these variables. Those with missing TUG time were also older (‘age’ p<0.001), weaker i.e. lower grip strength (p<0.001), inactive (‘ipaqkcal’ p<0.001), have poor cognitive score (‘mmse_errors’ p<0.001) and higher mean CRT values (‘logCRTmeantot’ p<0.001). P-values refer to t-test or Wilcox test.

# Figure S3. Margin plots between physical activity and other participant characteristics at Wave 1 to assess MAR assumption


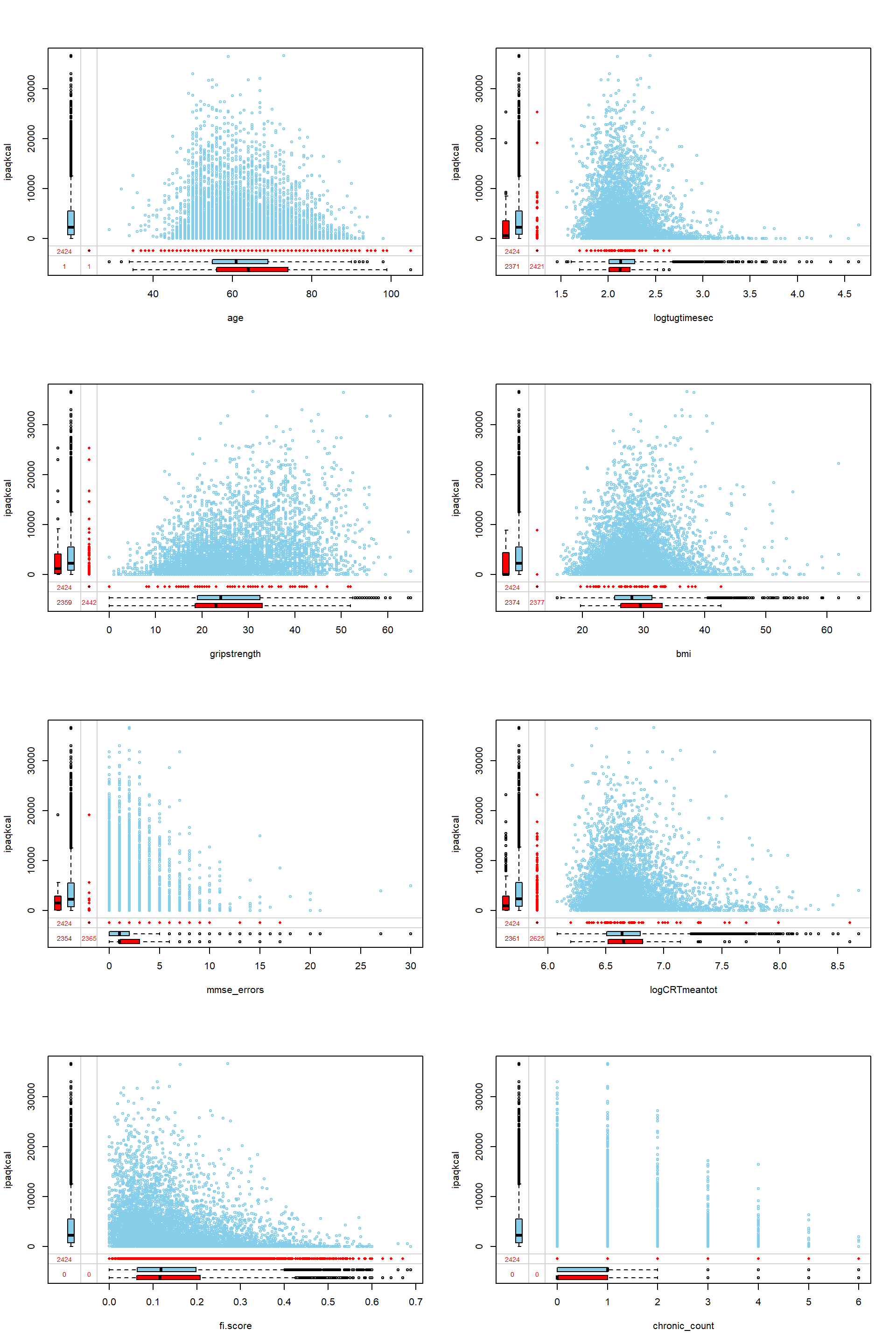


At Wave 1, 2424 participants were missing physical activity (‘ipaqkcal’) data. The distribution of ‘ipaqkcal’ was significantly lower for participants with values missing on TUG time, BMI, grip strength, the number of MMSE errors and mean CRT (left red boxes) compared to participants where these variables were present (left blue boxes), suggesting that the missingness of physical activity might be dependent on these variables. Those with missing ‘ipaqkcal’ were also older (‘age’ p<0.001). P-values refer to t-test or Wilcox test.

# Table S4. Baseline characteristics of participants followed-up versus participants lost to follow-up

| Mean (SD) or N (%),  unless specified | **Followed-up** | **Lost to follow-up** | **p-value*** |
| --- | --- | --- | --- |
| Total | 7493 (88.1%) | 1011 (11.9%) |  |
| Age | 62.8 (9.9) | 63.6 (10.7) | 0.02 |
| Sex (Female) | 4054 (55.6 %) | 579 (57.3 %) | 0.35 |
| Education level  Primary | 2469 (33.9 %) | 403 (39.9 %) | <0.001 |
| Secondary | 2983 (40.9 %) | 380 (37.6 %) |  |
| Tertiary | 1617 (22.2 %) | 179 (17.7 %) |  |
| Married | 5170 (71 %) | 709 (70.1 %) | 0.61 |
| Residential area  Rural | 3501 (48.1 %) | 498 (49.3 %) | 0.69 |
| Another city | 2056 (28.2 %) | 272 (26.9 %) |  |
| Dublin | 1728 (23.7 %) | 241 (23.8 %) |  |
| Lives alone | 1510 (20.7 %) | 221 (21.9 %) | 0.43 |
| Occupation type  Manual | 1931 (26.5 %) | 328 (32.4 %) | <0.001 |
| Non-manual | 5354 (73.5 %) | 683 (67.6 %) |  |
| BMI | 28.7 (5.1) | 28.7 (5) | 0.96 |
| Current smoker | 1264 (17.4 %) | 238 (23.5 %) | <0.001 |
| Drinking above the weekly limit† | 402 (5.5 %) | 42 (4.2 %) | <0.001 |
| Depressed | 674 (9.3 %) | 111 (11 %) | 0.01 |
| Chronic diseases (≥2) | 1295 (17.8 %) | 159 (15.7 %) | 0.12 |
| Cardiovascular diseases (≥3) | 848 (11.6 %) | 109 (10.8 %) | 0.45 |
| MMSE errors, median (IQR) | 1 (0-2) | 2 (0-4) | <0.001 |
| Accident and emergency (ever) | 1077 (14.8 %) | 142 (14 %) | 0.57 |
| Overnight hospitalisation (ever) | 905 (12.4 %) | 122 (12.1 %) | 0.79 |
| Attended health assessment centre | 4927 (67.6 %) | 290 (28.7 %) | <0.001 |
| FI score, median (IQR) | 0.12 (0.06-0.2) | 0.12 (0.06-0.19) | 0.57 |
| FP count, median (IQR) | 0 (0-1) | 1 (0-2) | 0.02 |
| Grip strength (kg) | 26.1 (9.9) | 26.1 (10.1) | 0.94 |
| TUG time (s) | 9 (3.6) | 9.5 (3.3) | 0.02 |
| IPAQ (kcal), median (IQR) | 2347 (830.9-5600.4) | 2096 (692.4-5074.3) | 0.03 |

*p-values refer to the Chi-square/Fisher’s exact tests for categorical variables or the t-test or Wilcox test for continuous variables

†The weekly limit on standard drinks (1/2 pint of beer or a glass of wine) according to the government guidelines (>21 for men; >14 for women)

Abbreviations: BMI: body mass index; MMSE: Mini-Mental State Examination; FI: frailty index; FP: frailty phenotype; TUG: Timed-Up-and-Go; IPAQ: International Physical Activity Questionnaire.

# Table S5. Exploratory Factor Analysis (EFA) results of the frailty phenotype and frailty index

|  | **Frailty Phenotype** | | **Frailty Index** | | | |
| --- | --- | --- | --- | --- | --- | --- |
| **Factor** | 1 | 2 | 1 | 2 | 3 | 4 |
| **Items** | - Grip strength - Physical activity - Walk time | - Energy - Weight loss | 10 items^a^ related to ADLs | - Hypertension - Angina - Heart attack - High cholesterol - Irregular heart rhythm - Other CVD - Polypharmacy | - Arthritis - Intrusive pain - Knee pain | 3 items^b^ related to hearing and vision impairment |
| **Covariance explained by factor** | 84% | 16% | 44% | 24% | 21% | 11% |
| **Total explained variability** | 31% | | 53% | | | |

^a^Difficulty preparing a hot meal; Difficulty with household chores; Difficulty shopping for groceries; Difficulty walking 100m; Difficulty climbing several flights of stairs; Difficulty climbing one flight of stairs; Difficulty reaching above shoulder height; Difficulty pushing or pulling large objects; Difficulty lifting or carrying weights 10 pounds or more; and Difficulty picking up coin from table.

^b^Poor self-rated vision; Poor self-rated hearing; and Difficulty following a conversation with one person.

**EFA results further details:** Kaiser-Meyer-Olkin (KMO) measure of sampling adequacy test showed that both instruments were suitable for FA with a KMO > 0.6 (0.69 and 0.61 for FI and FP, respectively) and significant Bartlett’s test (p<0.001). The EFA identified a 2- and 4-factor structure for the FP and FI, respectively (Table S5).

# Figure S4. Factor plot of a correlated 2-factor Confirmatory Factor Analysis (CFA) model of Frailty Phenotype (FP)

**
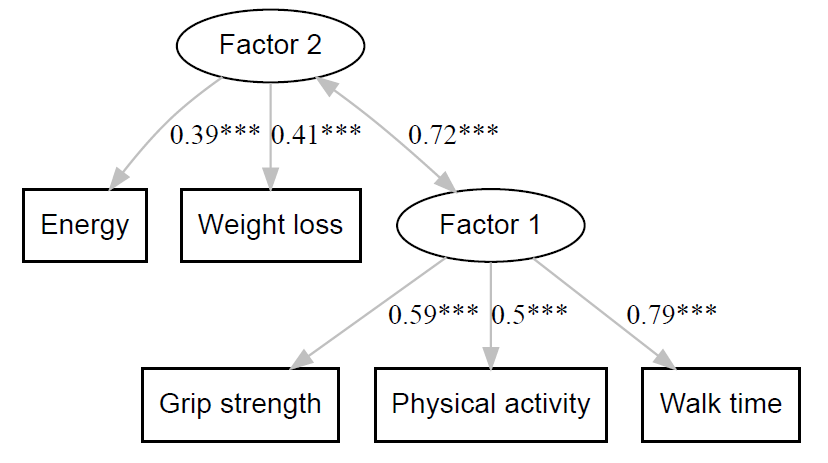
**

Numbers by the arrows represent coefficients, i.e. factor loadings, of the CFA model. Double arrows represent correlations among the latent factors. Coefficient significance are included in the form of significant stars where *p≤0.05, **p≤0.01, and ***p≤0.001.

**Fit statistics: CFI=0.996; TLI=0.991; RMSEA=0.014**

Abbreviations: CFI=comparative fit index; TLI=Tucker-Lewis Index; RMSEA=root mean square error of approximation

# Figure S5. Factor plot of a correlated 4-factor Confirmatory Factor Analysis (CFA) model of Frailty Index (FI)


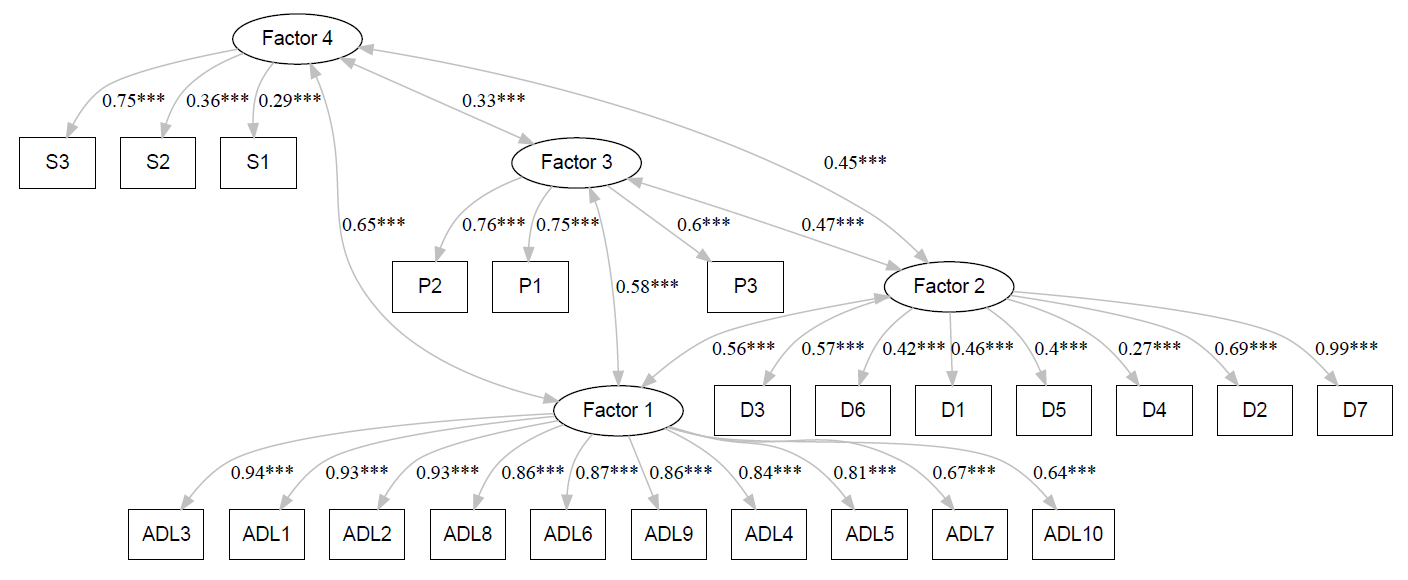


Key: ADL1=Difficulty preparing a hot meal; ADL2=Difficulty with household chores; ADL3=Difficulty shopping for groceries; ADL4=Difficulty walking 100m; ADL5=Difficulty climbing several flights of stairs; ADL6=Difficulty climbing one flight of stairs; ADL7=Difficulty reaching above shoulder height; ADL8=Difficulty pushing or pulling large objects; ADL9=Difficulty lifting or carrying weights 10 pounds or more; ADL10=Difficulty picking up coin from table; S1=Poor self-rated vision; S2=Poor self-rated hearing; S3=Difficulty following a conversation with one person; D1=Hypertension; D2=Angina; D3=Heart attack; D4=High cholesterol; D5=Irregular heart rhythm; D6=Other CVD; D7=Polypharmacy; P1=Arthritis; P2=Intrusive pain; P3=Knee pain

Numbers by the arrows represent coefficients, i.e. factor loadings, of the CFA model. Double arrows represent correlations among the latent factors. Coefficient significance are included in the form of significant stars where *p≤0.05, **p≤0.01, and ***p≤0.001.

**Fit statistics: CFI=0.974; TLI=0.970; RMSEA=0.039**

Abbreviations: ADL=activities of daily living; CFI=comparative fit index; D=disease; P=pain; RMSEA=root mean square error of approximation; S=sensory; TLI=Tucker-Lewis Index

# Figure S6. Distribution of the change in frailty phenotype count (A) and frailty index score (B) between Waves 1 and 2


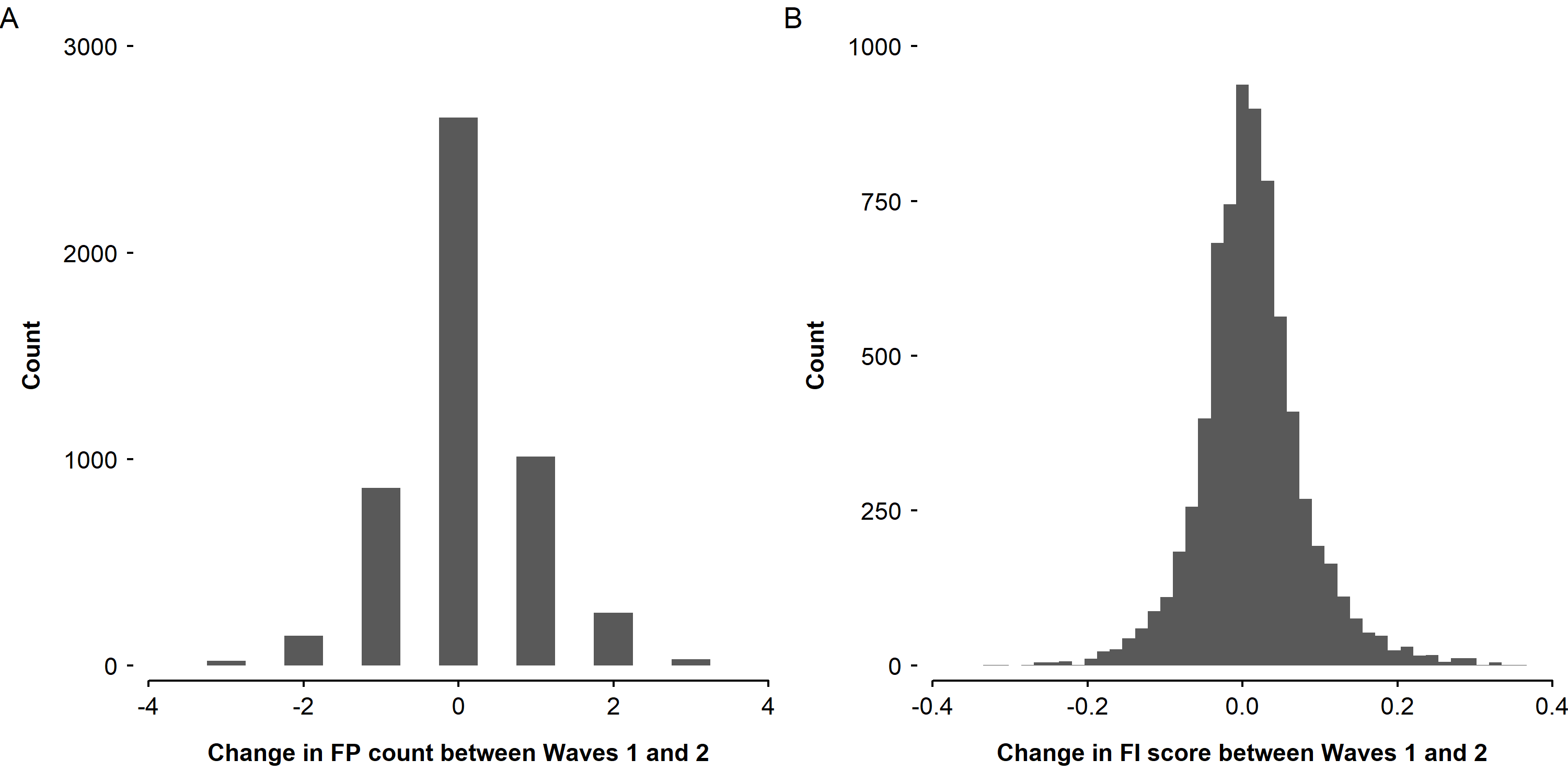


# Figure S7. Bland-Altman plot for the frailty index score


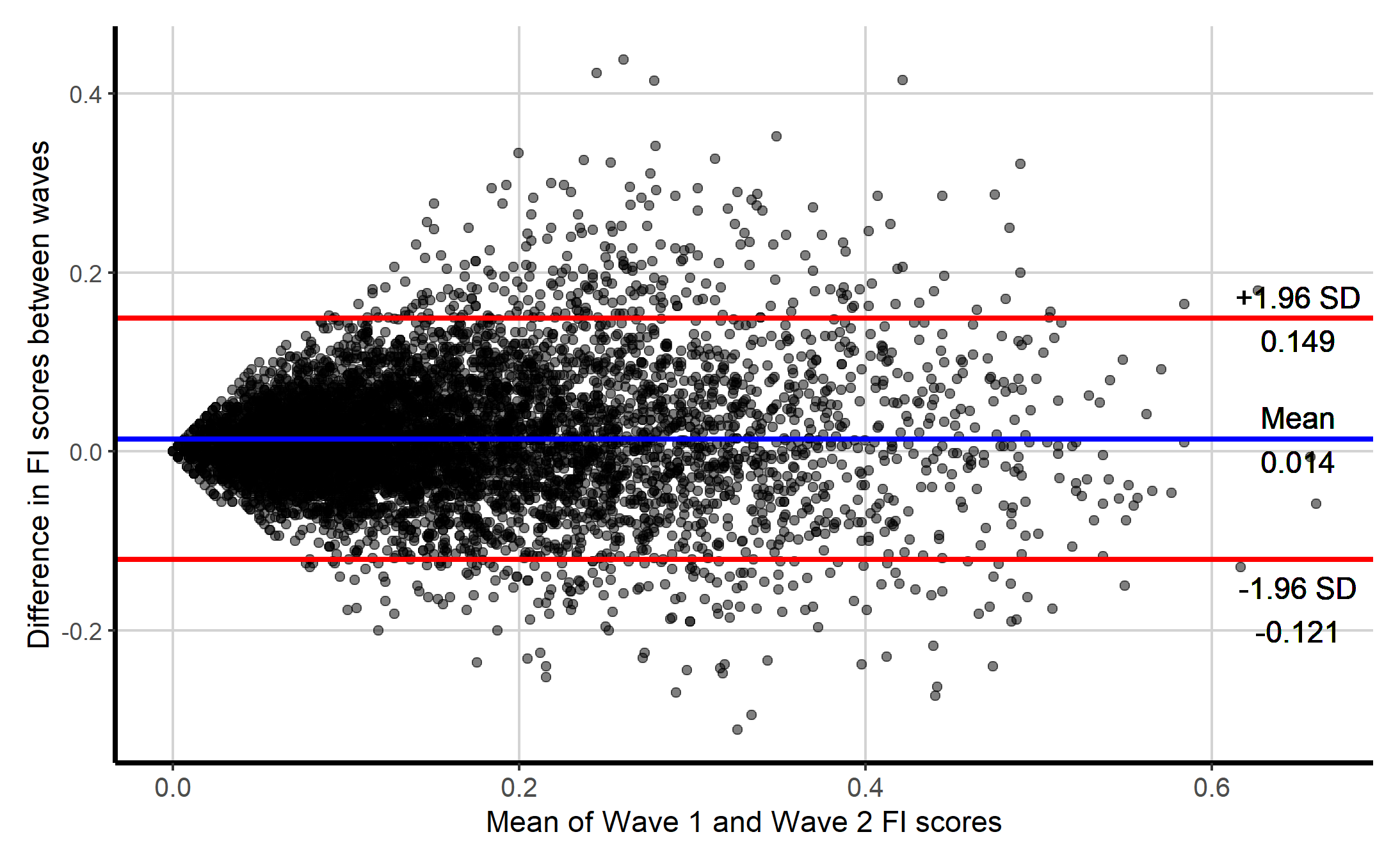


# Table S6. Average changes in frailty phenotype count and frailty index score between Waves 1 and 2 by level of change in self-reported health status over the same period

| **Level of change in self-reported health (SRH) status** | **N** | **Mean (SD) change in FP** | **Mean (SD) change in FI** |
| --- | --- | --- | --- |
| 2 units or more decline in SRH | 333 | 0.250 (1.192) | 0.059 (0.014) |
| 1 unit decline in SRH | 1471 | 0.311 (1.230) | 0.050 (0.040) |
| No change | 3369 | 0.106 (1.268) | 0.015 (0.044) |
| 1 unit improvement in SRH | 1685 | -0.051 (1.199) | -0.004 (0.037) |
| 2 units or more improvement in SRH | 348 | -0.100 (0.995) | -0.018 (0.015) |

Data used to calculate the MIC is shown in grey boxes.

Table S6 shows mean changes in the FP and FI between Waves 1 and 2 by the level of change in self-reported health status over the same period. Compared to baseline, 3369 participants reported no change self-reported health status at Wave 2, whilst 333 participants worsened by 2 or more units. The 3369 participants with no change in self-reported health had a mean change of FP and FI by 0.106 and 0.015, respectively, whilst those who experienced a 2 unit decline in SRH had a mean change of 0.250 and 0.059, respectively. Using the minimally important change (MIC) definition of 2 or more units decline in SRH, MIC is calculated as the difference in these means: 0.144 (i.e. 0.250-0.106) and 0.044 (i.e. 0.059-0.015) for the FP and FI, respectively. (Note that the magnitude of the MIC for FP was smaller in manuscript, which report the data obtained from multiple imputation.) When using an alternative definition of MIC, 1 unit decline in SRH, the calculation would be: 0.205 (i.e. 0.311-0.106) and 0.035 (i.e. 0.050-0.015), respectively.

# Table S7. Discrimination of the frailty phenotype and frailty index for recurrent falls and overnight hospital stay and all-cause mortality

|  | **Frailty Phenotype**  **AUC (95% CI)** | **Frailty Index**  **AUC (95% CI)** |
| --- | --- | --- |
| Falls |  |  |
| Wave 1 | 0.646 (0.642 - 0.650) | 0.687 (0.662 - 0.711) |
| Wave 2 | 0.643 (0.641 - 0.646) | 0.686 (0.662 - 0.711) |
| Mean of Wave 1 and 2 | 0.651 (0.648 - 0.654) | 0.693 (0.669 - 0.718) |
| Hospitalisation |  |  |
| Wave 1 | 0.700 (0.692 - 0.708) | 0.764 (0.732 - 0.796) |
| Wave 2 | 0.726 (0.723 - 0.731) | 0.778 (0.747 - 0.809) |
| Mean of Wave 1 and 2 | 0.734 (0.729 - 0.739) | 0.784 (0.753 - 0.814) |
| Mortality |  |  |
| Wave 1 | 0.840 (0.838 - 0.841) | 0.840 (0.823 - 0.857) |
| Wave 2 | 0.850 (0.848 - 0.851) | 0.847 (0.830 - 0.863) |
| Mean of Wave 1 and 2 | 0.849 (0.847 - 0.849) | 0.844 (0.828 - 0.861) |

Note: AUCs were computed using logistic regression models of the association between the predictors (continuous FI and FP separately) and outcomes adjusted for age, sex, education level, social class, marital status, smoking status, and alcohol drinking frequency at baseline.

Abbreviation: AUC: area under the receiver operating characteristic curve

# References

1. Fried LP, Tangen CM, Walston J *et al.* Frailty in Older Adults Evidence for a Phenotype. *J Gerontol Ser A* 2001;**56**:M146–57.

2. O’Halloran AM, Laird EJ, Feeney J *et al.* Circulating Micronutrient Biomarkers Are Associated With 3 Measures of Frailty: Evidence From the Irish Longitudinal Study on Ageing. *J Am Med Dir Assoc* 2019:S1525861019304979.

3. Wong TY, Massa MS, O’Halloran AM *et al.* Cardiovascular risk factors and frailty in a cross-sectional study of older people: implications for prevention. *Age Ageing* 2018;**47**:714–20.

4. Terwee CB, Bot SDM, De Boer MR *et al.* Quality criteria were proposed for measurement properties of health status questionnaires. *J Clin Epidemiol* 2007;**60**:34–42.

5. Prinsen C a. C, Mokkink LB, Bouter LM *et al.* COSMIN guideline for systematic reviews of patient-reported outcome measures. *Qual Life Res Int J Qual Life Asp Treat Care Rehabil* 2018;**27**:1147–57.

6. Nolte S, Coon C, Hudgens S *et al.* Psychometric evaluation of the PROMIS® Depression Item Bank: an illustration of classical test theory methods. *J Patient-Rep Outcomes* 2019;**3**:46.

7. Kottner J, Audigé L, Brorson S *et al.* Guidelines for Reporting Reliability and Agreement Studies (GRRAS) were proposed. *J Clin Epidemiol* 2011;**64**:96–106.

8. Streiner DL, Norman GR, Cairney J. Reliability. *Health Measurement Scales: A Practical Guide to Their Development and Use*. Fifth edition. Oxford: Oxford University Press, 2015.

9. Davidson M, Keating J. Patient-reported outcome measures (PROMs): how should I interpret reports of measurement properties? A practical guide for clinicians and researchers who are not biostatisticians. *Br J Sports Med* 2014;**48**:792–6.

10. de Vet HCW, Terwee CB. The minimal detectable change should not replace the minimal important difference. *J Clin Epidemiol* 2010;**63**:804–5.

11. McGlothlin AE, Lewis RJ. Minimal Clinically Important Difference: Defining What Really Matters to Patients. *JAMA* 2014;**312**:1342.

12. Tabachnick BG, Fidell LS. *Using Multivariate Statistics*. 6th ed. United States of America: Pearson, 2013.

13. Kaiser HF. A second generation little jiffy. *Psychometrika* 1970;**35**:401–15.

14. Bartlett MS. The effect of standardization on a χ2 approximation in factor analysis. *Biometrika* 1951;**38**:337–44.

15. Horn JL. A rationale and test for the number of factors in factor analysis. *Psychometrika* 1965;**30**:179–85.

16. Yong AG, Pearce S. A Beginner’s Guide to Factor Analysis: Focusing on Exploratory Factor Analysis. *Tutor Quant Methods Psychol* 2013;**9**:79–94.

17. Costello AB, Osborne J. Best practices in exploratory factor analysis: four recommendations for getting the most from your analysis. *Pract Assess Res Eval* 2005;**10**, DOI: 10.7275/JYJ1-4868.

18. Matsunaga M. How to factor-analyze your data right: do’s, don’ts, and how-to’s. *Int J Psychol Res* 2010;**3**:97–110.

19. Field A. *Discovering Statistics Using SPSS*. 4th ed. London: SAGE, 2013.

20. Norman GR, Streiner DL. *Biostatistics: The Bare Essentials*. PMPH USA (BC Decker), 2008.

21. Bentler PM. Comparative fit indexes in structural models. *Psychol Bull* 1990;**107**:238–46.

22. Tucker LR, Lewis C. A reliability coefficient for maximum likelihood factor analysis. *Psychometrika* 1973;**38**:1–10.

23. Steiger JH. Structural Model Evaluation and Modification: An Interval Estimation Approach. *Multivar Behav Res* 1990;**25**:173–80.

24. Hu L, Bentler PM. Cutoff criteria for fit indexes in covariance structure analysis: Conventional criteria versus new alternatives. *Struct Equ Model Multidiscip J* 1999;**6**:1–55.

25. Browne MW, Cudeck R. Alternative Ways of Assessing Model Fit. *Sociol Methods Res* 2016, DOI: 10.1177/0049124192021002005.
